# Is metformin a possible treatment for diabetic neuropathy?

**DOI:** 10.1111/1753-0407.13310

**Published:** 2022-09-18

**Authors:** Juechun Wei, Yanling Wei, Meiyan Huang, Peng Wang, Shushan Jia

**Affiliations:** ^1^ The Second Medical College Binzhou Medical University Yantai China; ^2^ Qingdao Dongheng Zhiyuan Automobile Service Co. LTD Qingdao China; ^3^ Yantai Affiliated Hospital of Binzhou Medical University Yantai China

**Keywords:** adenosine monophosphate‐activated protein kinase (AMPK), diabetic neuropathy, metformin, pain, vitamin B12, 二甲双胍, 糖尿病性神经病变, 疼痛, 维生素B12, 腺苷酸激活蛋白激酶(AMPK)

## Abstract

Metformin is a hypoglycemic drug widely used in the treatment of type 2 diabetes. It has been proven to have analgesic and neuroprotective effects. Metformin can reverse pain in rodents, such as diabetic neuropathic pain, neuropathic pain caused by chemotherapy drugs, inflammatory pain and pain caused by surgical incision. In clinical use, however, metformin is associated with reduced plasma vitamin B12 levels, which can further neuropathy. In rodent diabetes models, metformin plays a neuroprotective and analgesic role by activating adenosine monophosphate‐activated protein kinase, clearing methylgloxal, reducing insulin resistance, and neuroinflammation. This paper also summarized the neurological adverse reactions of metformin in diabetic patients. In addition, whether metformin has sexual dimorphism needs further study.

## Graphical abstract

**Highlights**

- Metformin has been shown to alleviate diabetic peripheral neuropathy.
- The neuroprotective and analgesic effects of metformin are related to the activation of AMPK, the clearance of MGO, and the reduction of insulin resistance and neuroinflamation.
- AMPK activation is critical for the analgesic effect of metformin.
- AMPK activation can attenuate inflammatory response and oxidative stress, regulate autophagy and regulate ion channels.

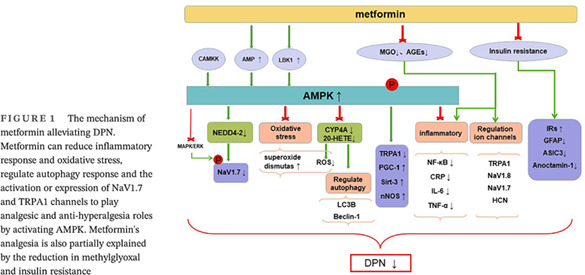

## 摘要

二甲双胍是一种广泛用于治疗2型糖尿病的降糖药物。它已被证明具有镇痛和神经保护作用。二甲双胍可以逆转啮齿类动物的疼痛，如糖尿病性神经痛、化疗药物引起的神经性疼痛、炎症性疼痛、手术切口引起的疼痛。然而，在临床使用中，二甲双胍与血浆维生素B12水平降低有关，这可能会进一步加剧神经病变。在啮齿动物糖尿病模型中，二甲双胍通过激活AMPK、清除MGO、降低胰岛素抵抗和神经炎症发挥神经保护和镇痛作用。本文还总结了二甲双胍对糖尿病患者的神经系统不良反应。此外，二甲双胍是否有两性区别还有待进一步研究。

## 1 INTRODUCTION

Diabetic peripheral neuropathy (DPN) is a common complication of diabetes. DPN is mainly characterized by sensory neuropathy but can also be accompanied by autonomic neuropathy and impaired motor function.^1^ About 30% DPN patients have pain symptoms, including tingling, burning, spontaneous pain, allodynia and hyperalgesia.^2^ However, the treatment of painful diabetic peripheral neuropathy (PDPN) has been unsatisfactory, and less than 30% PDPN patients can obtain satisfactory analgesic through therapy.^3^ Metformin is an antihyperglycemic drug widely used in patients with type 2 diabetes mellitus (T2DM) and has been reported to have analgesic potential.^4^ Studies have shown that metformin can relieve neuropathic pain caused by diabetes^5^, ^6^ or chemotherapeutic drugs,^7^, ^8^ low back pain,^9^, ^10^ osteoarthritis,^11^, ^12^ postoperative pain^13^, ^14^ and inflammatory pain^15^, ^16^ through activating adenosine 5′monophosphate‐activated protein kinase (AMPK). Metformin also has the ability to remove methylglyoxal (MGO),^17^ a metabolite associated with PDPN.^18^ However, there is an interesting phenomenon in the clinical application of metformin——it may reduce serum vitamin B12 level, which may aggravate nerve injury.^19^, ^20^

Here, we reviewed the basic and clinical studies and summarized research status and mechanism of metformin in treating DPN. Data strongly suggested that metformin may be a promising agent in alleviating DPN. However, more clinical studies are needed to evaluate its efficacy and safety.

## 2 EFFECTS AND MECHANISMS OF METFORMIN ON PDPN

**FIGURE 1:**
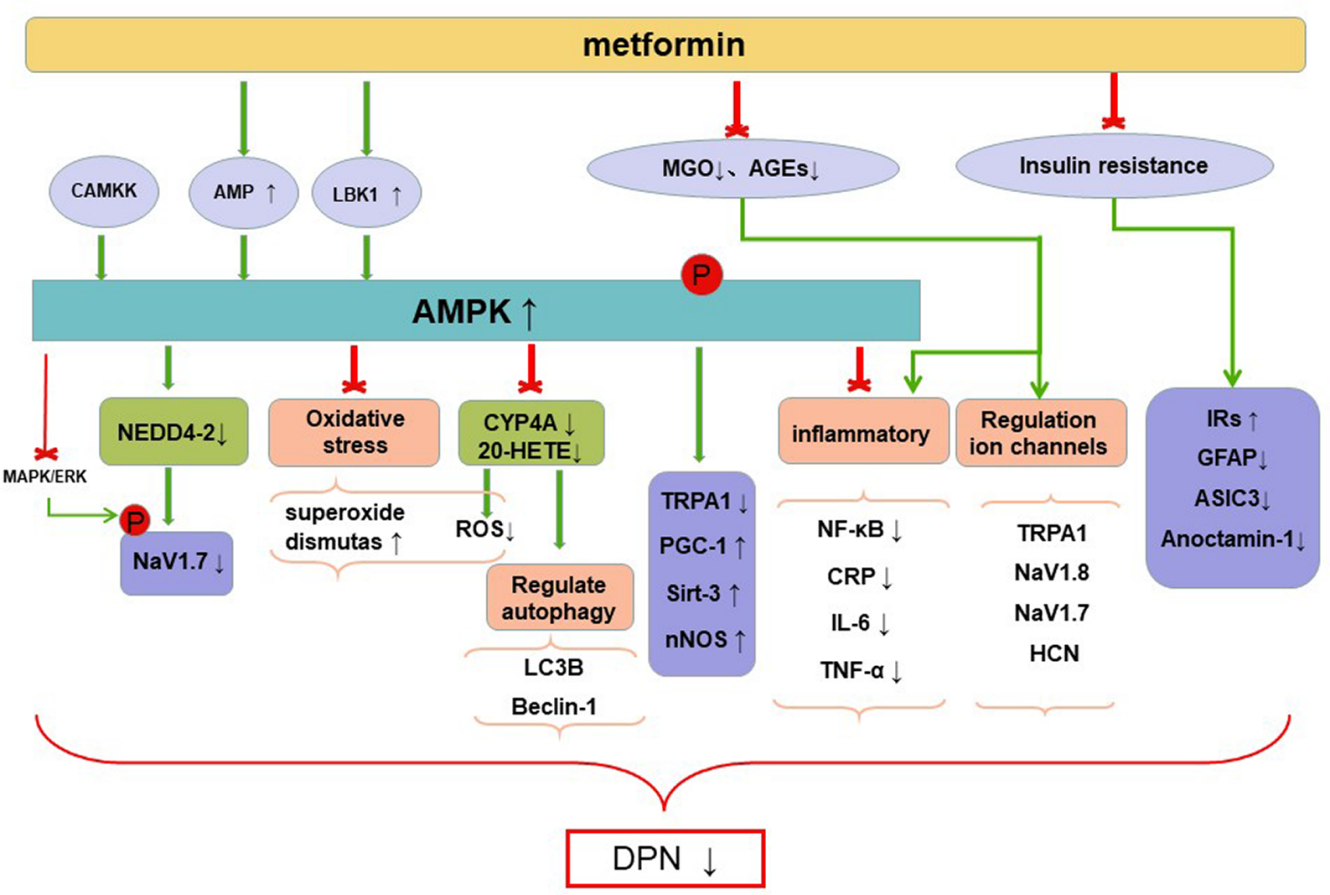
The mechanism of metformin alleviating DPN. Metformin can reduce inflammatory response and oxidative stress, regulate autophagy response and the activation or expression of NaV1.7 and TRPA1 channels to play analgesic and anti‐hyperalgesia roles by activating AMPK. Metformin's analgesia is also partially explained by the reduction in methylglyoxal and insulin resistance

Metformin acts through multiple pathways, including inhibiting mitochondrial respiration and gluconeogenesis, activating AMPK, increasing insulin sensitivity, antagonizing the effects of glucagon, and increasing fatty acid oxidation.^21^ It was previously thought that hyperglycemia is the main cause of PDPN,^22^ but this view has recently been challenged. In one study, feeding a 15% fructose solution to rats for 16 weeks induced generalized tactile pain and hyperalgesia with no significant elevation of blood glucose levels,^23^ suggesting that the effect of fructose on pain may be due to insulin resistance rather than hyperglycemia.^24^ Allodynia and hyperalgesia in fructose‐treated rats can be reversed by metformin, suggesting that its antihyperalgesia effect was not entirely dependent on hypoglycemic effect. Metformin also reversed the reduction of mechanical withdrawal threshold without changing in plasma glucose and insulin in high‐fat diet/streptozotocin rats.^25^ The mechanism of metformin alleviating DPN is shown in the Figure 1 and studies involved are shown in Table 1.

**TABLE 1:** Summary of studies about the neuroprotective effect of metformin in diabetic neuropathy models.

Study	Mechanisms of metformin	Models	Doses, time, and administration route of metformin	Effects of metformin
Hasanvand^26^	AMPK/IL‐6, CRP, TNF‐α	STZ‐induced DN rats	metformin (300 mg/kg/d, gavage.) from 3 days after STZ injection until the end of the study	Decreased blood glucose; restored MNCV; activated AMPK; reduced IL‐6, CRP, and TNF‐α
Cao^5^	AMPK/NF‐κB	STZ‐induced DN rats	metformin (200 mg/kg/d, i.p.) for 6 days from the third week after STZ injection	Attenuated mechanical allodynia; activated AMPK; reduced NF‐κB
Haddad^27^	AMPK/CYP4A/20‐HETE	MKR nonobese T2DM male mice	Metformin (150 mg/kg/d, i.p.) for 13 weeks from 10 weeks after diabetes onset	Decreased blood glucose; corrected neural sensory and motor abnormalities; blunted oxidative stress and peripheral nerve injury; alleviated autophagy protein alterations triggered by hyperglycemia;
Ma^28^	AMPK/oxidative stress	STZ‐induced PDPN rats	Metformin (300 mg/kg/d, i.P.) from 21 days after STZ injection until the end of the study	inhibited mechanical hyperalgesia; increased paw withdrawal latency to heat and cold; activated AMPK and AMPK target genes (PGC‐1α, Sirt‐3, and nNOS); decreased malondialdehyde and glycation end‐products; increased superoxide dismutase
García^23^	Rescued insulin resistance	Fructose‐induced insulin resistance neuropathic pain rats	Metformin (50 mg/kg/d, po) for 4 weeks starting at 12 weeks after chronic fructose administration	Reversed fructose‐induced tactile allodynia; reduced fructose‐induced increased expression of GFAP; reversed fructose‐induced downregulation of the insulin receptor β protein expression
Byrne^25^	/	High‐fat diet/STZ induced diabetes rats	Metformin (200 mg/kg/d, po) from day 4 until day 40 after induced by STZ	Prevented reduction of mechanical withdrawal thresholds in high‐fat diet/STZ rats;
Wang^29^	AMPK/TRPA1	STZ‐induced DN mice	metformin (10 ml; 50 mmol/L in saline, subcutaneous injection) for short‐term treatment; metformin (250 mg/kg; 62.5 mg/ml in saline i.p.) for long‐term treatment	Inhibited high‐glucose‐promoted TRPA1 activation; alleviated mechanical allodynia in diabetic mice
Huang^30^	AMPK/MGO	STZ‐induced DN rats	Metformin was incubated at 1200 mmol/L with methyl glyoxal (400 mmol/L) at 37°C for 3 h; metformin (250 mg/kg, subcutaneous injection once) at 4 weeks after STZ	Reduced the free MGO level by 99.4%; reduced MGO‐induced acute flinching responses; blocked STZ‐induced mechanical allodynia

Abbreviations: 20‐HETE, 20‐hydroxyeicosatetraenoic acid; AMPK, adenosine monophosphate protein kinase; CRP, C‐reactive protein; CYP, cytochrome P450; DN, diabetic neuropathy; GFAP, glial fibrillary acidic protein; IL‐6, interleukin‐6; MGO, methylglyoxal; MNCV, Motor nerve conduction velocities; NF‐κB, nuclear factor‐kappa B; PDPN, painful diabetic peripheral neuropathy; STZ, streptozotocin; T2DM, type 2 diabetes mellitus; TNF‐α, tumor necrosis factor‐α; TRPA1, transient receptor potential ankyrin 1.

### 2.1 Activation of AMPK pathway

AMPK is a ubiquitous energy‐sensitive kinase consisting of two regulatory subunits (β and γ) and one catalytic subunit (α).^31^ When AMP/ATP ratio increases, AMP binding to γ‐subunit and the phosphorylation at Thr172 in the α‐subunit by Ca21/calmodulin‐dependent protein kinase or liver kinase B1 (LKB1) leads to indirect activation.^32^, ^33^ AMPK also can be activated directly by phosphorylation of α‐subunit by certain drugs such as O304.^10^ Metformin activates AMPK mainly by inhibiting mitochondrial respiratory chain complex 1 and ATP production^34^ and also acts in a LKB1‐dependent fashion.^35^ Impairment of AMPK is associated with a number of pathological conditions, including obesity, exercise or metabolic syndrome, and inflammation.^36^ Recently, studies have shown that the downregulated AMPK is closely related to pain symptoms and metformin can relieve pain by activating AMPK.^3^, ^5^, ^10^, ^36^

#### 2.1.1 Inhibit inflammatory response and oxidative stress

Metformin activates AMPK to inhibit cytokines and reduce systemic inflammation. Peripheral nerve inflammation is one of the mechanisms of DPN.^18^ Metformin increased the expression of phosphorylated AMPK (P‐AMPK, the activated state of AMPK) in dorsal root ganglia (DRG) cells, which decreased in diabetes.^26^ Compared with controls (saline), metformin reduced the serum levels of markers for inflammatory response such as C‐reactive protein, interleukin‐6, and tumor necrosis factor‐α.^26^ Metformin increased motor nerve conduction velocity, an indicator of diabetic neuropathy,^37^ in STZ‐induced diabetic mice.^26^ These results suggested metformin may play a neuroprotective role by activating AMPK to reduce inflammation. The nuclear factor NF‐κB pathway is a classic proinflammatory signaling pathway^38^ and also promotes DPN. Metformin attenuated mechanical allodynia through downregulating NF‐κB expression in L4‐6 DRG neurons, and the expression of NF‐κB was controlled by P‐AMPK.^5^

Inhibition of oxidative stress through activating AMPK may be a beneficial effect of metformin. Oxidative stress is an important part in the development of diabetes complications.^39^ Increased lipid peroxidation such as malondialdehyde and glycation end products and decreased superoxide dismutase activity, which can be reversed by metformin, were observed in STZ‐induced DPN rats.^28^ AMPK is closely related to oxidative stress, and its activation can ameliorate oxidative stress and protect cells from oxidative stress damage.^36^, ^40^, ^41^ Metformin activates AMPK and increases the expression of AMPK target proteins (PGC‐1α, Sirt3, and nNOS) associated with DN.^28^ Metformin inhibited oxidative stress by activating AMPK, which may partly explain its effect on relieving hyperalgesia (including mechanical hyperalgesia, hot allodynia, and cold allodynia).^28^ CYP4A is a subgroup of cytochrome P450 (CYP) enzyme family, CYP4A ω‐hydroxylase converts arachidonic acid to 20‐hydroxyeicosatetraenoic acid (20‐HETE).^42^ CYP enzyme is the main source of reactive oxygen species (ROS), and 20‐HETE mediates inflammation and oxidation. The expression lever of CYP4A, 20‐HETE, and ROS was increased in sciatic nerve tissues of MKR nonobese T2DM mice, both HET0016 (a specific 20‐HETE synthase and/or CYP4A inhibitor) and metformin reduced ROS and corrected neural sensory and motor abnormalities of T2DM mice.^27^ In addition, metformin reduced the level of 20‐HETE, and HET0016 restored the expression of P‐AMPK, which suggested that CYP4A/20‐HETE/AMPK mediate nerve damage through oxidative stress and an interaction existed between them.

#### 2.1.2 Regulate autophagy

Beclin‐1 and LC3B, the key proteins of the autophagic response, significantly increased in the sciatic nerve of mice with diabetes and were also affected by CYP4A/20‐HETE/AMPK.^27^ A cell‐level study showed that high glucose increased autophagosome and LC3B in PC12 cells.^43^ Metformin can alleviate autophagy (decreased beclin‐1 and LC3B expression in sciatic nerve) by activating CYP4A/20‐HETE/AMPK pathological axis, saving peripheral nerve injury in diabetic mice.^27^ However, there are conflicting conclusions regarding autophagy in DN. The level of beclin‐1 in sciatic nerve decreased in STZ‐induced diabetic rats, and P‐AMPK enhanced autophagy response by promoting autophagosome formation.^44^ Autophagy is a cellular process in which senescent or damaged organelles and proteins in the autophagosome are sequestered to recover their products and is also involved in the elimination of cells that undergo classic type 1 programmed cell death. Therefore, autophagy is generally considered to be a protective cellular response against various stressors and daily wear and tear. On the other hand, autophagy may also lead to a nonapoptotic cell death called type 2 programmed cell death.^45^ We hypothesize that the conflicting conclusions about autophagy may be due to the different models used in the studies.

#### 2.1.3 Regulate ion channel

Voltage‐gated sodium channel Nav1.7 is selectively expressed in sensory and autonomic neurons and associated with excitability of nociceptive neurons.^46^ Pain can be relieved by downregulating NaV1.7 expression or using NaV1.7 blockers. Metformin enhanced the function of neuronal precursor cell expressed developmentally downregulated‐4 type 2 (NEDD4‐2, a ubiquitin protein ligase) by activating AMPK, then induced a decreased expression of NaV1.7, and ultimately reduced the excitability of nociceptive neurons.^6^

In addition to expression level, the gated properties of NaV1.7 also affect the excitability of nociceptive neurons. Mitogen‐activated protein kinase (MAPK) family is a pivotal signaling system with at least four pathways. Among them, the MAPK/ERK (extracellular‐regulated kinase) pathway has been implicated in many cellular functions (including migration, differentiation, apoptosis, and senescence) and pathologies including pain.^47^ NaV1.7 is activated by directly phosphorylation by pERK1/2 (a phosphorylated ERK), and inhibition of pERK1/2 reduces the excitability of DRG neurons.^48^ The upstream activation mechanism of ERK signaling pathway may be related to the inactivation of AMPK,^49^ so metformin can reduce cell excitability by activating AMPK. Although this mechanism has not been directly validated in a diabetes model, it has been demonstrated in a model of neuropathic pain induced by chronic compression of the dorsal root ganglion and high‐fat diet and in cell experiments.

The transient receptor potential ankyrin 1 (TRPA1) channel is a pain sensor mainly expressed in primary sensory neurons,^50^ which can be activated by accumulated methylglyoxal in hyperglycemia patients to facilitate PDPN.^51^ Studies have shown that TRPA1 can also be regulated by AMPK.^29^ TRPA1 is a transmembrane protein whose activity is determined by the expression level of plasma membrane. Metformin reduced the level of membrane‐associated TRPA1 in DRG neurons cultured with high glucose and alleviated mechanical allodynia in diabetic mice.^29^

Metformin can also treat other types pain by activating AMPK. In mouse models of postoperative pain, activated AMPK negatively regulated mammalian target of rapamycin (mTOR) signaling pathway, preventing acute pain from transitioning to chronic pain.^14^ Activation of the mTOR/p70S6K pathway is involved in chronic pain caused by nerve injury or inflammation.^52^ AMPK inhibits the mTOR/p70S6K signaling pathway, thereby alleviating painful radiculopathies in lumbar disc herniation rat model and acute pain induced by oxaliplatin in rat models,^9^ which may be associated with inhibition of herniation plasticity.^53^

### 2.2 Remove methylglyoxal

MGO is a reactive carbonyl compound produced by a nonenzymatic pathway of glucose metabolism and it has been reported to be associated with PDPN.^54^ Methylglyoxal spontaneously reacts with biopolymers to form advanced glycation end products (AGEs), and it mainly degraded by glyoxalase system, where glyoxalase I (Glo1) is the rate‐limiting enzyme.^55^ Studies have shown that increased AGEs production and decreased GloI activity are conducive to the development of PDPN.^56^ Methylglyoxal may induce pain symptoms through oxidative stress,^57^ inflammatory stimulation^58^ and regulation ion channels (such as TRPA1,^59^, ^60^ NaV1.8,^30^, ^61^ and NaV1.7^62^) and hyperpolarization–activated cyclic nucleotide‐gated channel.^63^

T2DM patients with metformin had lower plasma methylglyoxal levels, despite similar blood sugar levels.^64^, ^65^ Metformin also reduced AGEs.^66^, ^67^ Therefore, metformin is considered a potential drug for the treatment of PDPN, which has been confirmed in basic experiments.^30^

Metformin can clear MGO in vivo and in vitro. When MGO was co‐incubated with metformin, free MGO levels were reduced by more than 90%. When the incubation solution was injected into the hind paw of mice, no mechanical pain was observed. Systemic injection of metformin can effectively inhibit the pain induced by claw injection of MGO and significantly block STZ‐induced mechanical pain.^30^ Intrathecal injection of metformin also significantly reduced the upregulated MGO in the dorsal horn and mechanical pain induced by bortezomib treatment, which may be related to RAGE/STAT3 signalling pathway.^68^ Metformin also reduced MGO‐induced apoptosis by inhibiting oxidative stress in vivo and in vitro.^69^ Taken together, the data suggested that metformin, as an MGO scavenger, may serve as a disease reliever in PDPN.

### 2.3 Reduce insulin resistance

In recent years, the conclusion that hyperglycemia is the main cause of diabetic neuropathy has been challenged by the research results. Evidence suggested that peripheral neuropathy is also present in normoglycemic patients with metabolic syndrome and in a progenitor state prior to the establishment of T2DM.^70^, ^71^ Insulin resistance (IR) is a condition in which the efficiency of insulin in promoting glucose uptake and utilization is reduced and in which the body compensates by producing too much insulin to maintain blood sugar stability, eventually leading to hyperinsulinemia.^72^

Evidence for IR affecting nociception is as follows: (1) Insulinemia itself or its consequences play a role in mechanistic nociceptive injury. Chronic fructose treatment increased plasma insulin level and caused hyperalgesia and abnormal tactile pain without a significant increase of blood glucose levels.^23^ Compared with the control group, mechanical pain thresholds and the serum insulin level were decreased in both hyperglycemic and normal‐glycemic rats after STZ treatment, but there was no statistical difference in blood glucose between two groups.^73^ (2) IR is associated with altered nociception. The homeostatic model assessment for insulin resistance index is an indicator of steady state beta cell function and insulin sensitivity, and its increase is associated with tactile allodynia and chemical hyperalgesia.^23^ (3) The effect on nociception may be because of insulin receptor expression or signal transduction related events rather than hyperglycemia. In the early course of nociceptive dysfunction, IR signal impairment in peripheral nerves was observed, which manifested decreased phosphorylation of insulin receptors and Akt protein.^74^, ^75^ Clinical trials have also shown that people with IR are significantly more likely to have pain.^70^, ^76^

Metformin, an insulin sensitizer, reversed tactile pain and downregulated insulin receptor β in fructose‐induced IR rats with neuropathic pain. Metformin reversed the upregulation of GFAP (astrocyte activation markers), ASIC3 (acid‐sensing ion channel 3), and anoctamin‐1(an ionic channel involved in the nociceptive processing) in DRG of fructose induced IR rats.^23^ The mechanism of metformin treating PDPN by reducing IR remains further studied.

## 3 ADVERSE REACTIONS IN CLINICAL APPLICATION OF METFORMIN

According to the current clinical experience and data of metformin, there are few serious safety concerns. Rare and mild side effects such as hypoglycemia, gastrointestinal side effects (typically nausea, vomiting, or diarrhea), impaired liver and kidney function, and heart failure can be effectively avoided by adjusting dosage and form.^77^ In addition, it is worth noting that metformin affected the status of vitamin B1, vitamin B12, vitamin D, folic acid, and magnesium and it interfered with the microbiome.^78^ Vitamin B12 deficiency can cause neurological dysfunction (such as peripheral and autonomic neuropathy, painful neuropathy) and accelerate the progression of DN.^79^ Therefore, we focused on the effects of metformin and vitamin B12 on neurological.

Since 1971, when malabsorption of vitamin B12 in diabetic patients with metformin was first reported,^80^ numerous clinical trials and meta‐analyses have confirmed the association between metformin and low vitamin B12 levels.^81^, ^82^, ^83^, ^84^, ^85^, ^86^, ^87^ Vitamin B12 is found in foods as a protein‐binding form and released under the action of gastric acid pepsin and trypsin after entering the digestive tract. Then the free vitamin B12 is bound by the intrinsic factor (IF, a glycosylated protein secreted by gastric parietal cells) forming IF‐vitamin B12 complex, which is eventually absorbed in the ileum.^88^ Metformin is the main cause of vitamin B12 deficiency in T2DM patients^78^ and may be caused by the following mechanisms: (1) bacterial overgrowth caused by changes in the motility of the small intestine, (2) reduced vitamin B12 absorption due to changes of IF levels, and (3) inhibition of calcium‐associated vitamin B12‐IF complex absorption in the terminal ileum.

Many clinical studies have investigated the relationship between metformin‐induced vitamin B12 deficiency and PDN, but they have reached inconsistent conclusions. In some cross‐sectional studies, T2DM patients were divided into metformin and metformin‐free groups based on metformin medication history. Results showed a higher incidence of vitamin B12 deficiency^86^, ^89^, ^90^, ^91^ and a higher incidence of neuropathy in the metformin group^89^, ^90^, ^91^ or no different than in the non‐metformin group.^86^ Other studies included only T2DM patients with a history of metformin use, dividing them into vitamin B12 deficient or normal groups based on their serum vitamin B12 concentrations. There was no difference in the incidence of neuropathy between the two groups in these studies.^92^, ^93^ There is also a study supporting that metformin‐treated T2DM patients have a lower prevalence of neuropathy than non‐metformin‐treated T2DM patients.^90^

In addition to these two grouping methods, researchers discussed the influence of metformin on DPN from other perspectives. We summarized these studies in Table 2. Russo et al found that 30% metformin‐treated T2DM patients had neuropathy, and there was no difference in vitamin B12 levels between the neuropathy group and the normal group.^97^ Khalaf reported a 29% incidence of vitamin B12 deficiency and a 46% incidence of neuropathy among T2DM patients with metformin, but vitamin B12 concentration was not associated with the incidence of peripheral neuropathy.^96^ Furthermore, a randomized, double‐blind, placebo‐controlled trial demonstrated that oral vitamin B12 (methyl cobalamin) 1000 μg/day for one year increased plasma vitamin B12 levels and improved all neurophysiological parameters, sudomotor function, pain score, and quality of life.^98^ This study involved 90 T2DM patients taking metformin for at least 4 years with DN, which indirectly demonstrates a link between metformin, vitamin B12 deficiency, and neuropathy. However, a meta‐analysis found no significant association between the risk of neuropathy and metformin use in diabetic patients, though a higher risk of vitamin B12 deficiency in patients with metformin.^81^ This result may be because of the heterogeneity of the included studies, such as inconsistencies of diagnostic criteria for neuropathy and metformin exposure time, so more exploration is needed on the relationship between metformin‐induced vitamin B12 deficiency and neuropathy.

**TABLE 2:** Clinical studies involving metformin, vitamin B12 deficiency, and diabetic neuropathy.

Study	Characteristics of patients	Duration of diabetes(years)	HbA1c levels(%)	Results of prevalence of vitamin B12 deficiency	Results of prevalence of neuropathy
Singh A K.^89^	T2DM patients were divided into metformin exposed group and nonmetformin exposed group	/	MET: 8.2 ± 1.02 NMET: 8.4 ± 0.81	MET:21.4% NMET: 5.7% *p* < .05	MET: 53.6% NMET: 25% (*p* < .05)
de Groot‐Kamphuis^90^	T2DM patients were divided into metformin exposed group and nonmetformin exposed group	MET: 12.1 (7.6–17.9) NMET: 17.9 (12.1–23.9)	/	MET: 14.1% NMET: 4.4% *p* < .05	MET: 17.4% NMET: 28.1% (*p* < .05)
Wile^91^	T2DM patients were divided into metformin exposed group and nonmetformin exposed group	MET: 5.5 ± 3.3 NMET: 4.7 ± 2.9	MET: 6.7 ± 1.0 NMET: 6.8 ± 1.1	MET:31% NMET: 3% *p* < .05	MET: 10% NMET: 5% (*p* < .05)
Hashem^94^	T2DM patients with DPN were divided into metformin exposed group and nonmetformin exposed group	MET: 5.9 ± 4.3 NMET: 3.9 ± 4.8	MET: 7.6 ± 1.1 NMET: 6.7 ± 1.0	MET:25% NMET: 3% *p* < .05	There was a significant inverse relationship between DPN severity and cobalamin level (r = −0.81,*p* < .05)
Aroda^86^	persons at high risk for T2DM were divided into metformin exposed group and nonmetformin exposed group	/	MET: 5.90 ± 0.64 NMET: 6.02 ± 0.7	MET: 37% NMET: 20% *p* < .05	MET: 9.7% NMET:9.9% (*p* > .05)
Roy^95^	T2DM patients without DPN were divided into metformin exposed group and nonmetformin exposed group	≤5 years	MET: 7 0.29 ± 0.28 NMET: 7 0.35 ± 0.35	MET: 306.31 ± 176.70 mg/dl NMET: 627.54 ± 168.32 mg/dl *p* < .01	MET: 54.28% NMET: 28.57% (*p* < .05)
Khalaf^96^	Metformin‐treated T2DM patients were divided into vitamin B12‐deficient and normal groups.	8.67 ± 5.98	9.8 ± 1.8	Prevalence of vitamin B12 deficiency was 29%	46% of all patients had peripheral neuropathy but vitamin B12 concentration was not associated with the incidence of peripheral neuropathy
Ahmed^92^	Metformin‐treated T2DM patients were divided into vitamin B12‐deficient and normal groups.	LVB: 12 (8.75–17) NVB: 9 (5–16)	LVB: 7.4 (6.3–9.6) NVB: 9.4 (7.5–11.2)	Prevalence of vitamin B12 deficiency was 28.1%	MET: 32.3% NMET: 36.8% (*p* > .05).
Biemans^93^	Metformin‐treated T2DM patients were divided into vitamin B12‐deficient and normal groups.	LVB: 9.3 ± 5.2 NVB: 8.5 ± 6.3	LVB: 7.3 ± 1.1 NVB: 7.4 ± 1.2	Prevalence of cobalamin deficiency was 28.1%; Prevalence of holoTCII deficiency was 3.9%	Neuropathy occurred more frequently in cobalamin‐deficient (26.7 vs. 21.8%,*p* > .05) and holoTCII‐deficient patients (29.4 vs. 20.6%,*p* > .05) compared with normal groups; however, the differences were not significant
Russo^97^	Metformin‐treated T2DM patients were divided into neuropathy and normal groups	T2DM with DPN: 13.6 ± 10.1 T2DM without DPN: 10.9 ± 8.6	T2DM with DPN: 7.7 ± 1.5 T2DM without DPN: 7.3 ± 1.4	T2DM with DPN: 621.2 ± 267.3 pg/ml T2DM without DPN: 598.9 ± 316.2 pg/ml, *p* > .01	Metformin treatment was associated with a mild decline in vitamin B12 levels, but not DPN

*Note*: Data are expressed as mean ± SD, median (25–75 percentile) or *n* (%).

Abbreviation: cobalamin, vitamin B12; DPN, diabetic peripheral neuropathy; holoTCII, holo‐transcobalamin; LVB, low vitamin B12; MET, metformin exposed group; NMET, nonmetformin exposed group; NVB, normal vitamin B12; T2DM, type 2 diabetes mellitus.

Anemia is also a potential consequence of vitamin B12 deficiency, which occurs much later than neuropathy.^89^ Anemia was not the primary objective of most studies, and metformin exposure was not long enough to reveal a significant association between metformin use and anemia. A long‐term study showed patients at high risk for diabetes who took metformin for 5 years had a significantly higher prevalence of anemia than those who did not.^86^ More long‐term studies are needed in the future to explain the association between anemia and metformin use.

## 4 SEX DIFFERENCES IN METFORMIN ON PAIN

A large body of studies have clearly suggested that men and women respond to pain differently, as well as to pharmacological pain interventions.^99^ There was no difference in the prevalence of diabetic neuropathy between different gender,^100^ but female may be a risk factor of PDPN.^101^, ^102^

In some mouse models of pain, the pain relief effect of metformin is also sexually dimorphic.^14^, ^103^ Metformin reduced spared nerve injury‐induced mechanical hypersensitivity and cold‐induced mechanical hypersensitivity and reversed the activation of microglia in the spinal cord in male mice instead of female mice.^103^ It was observed that metformin reduced incision‐induced mechanical hypersensitivity and blocked hyperalgesia initiation only in male mice.^14^ Sex hormones may influence the effect of metformin on neuropathic pain. Metformin is a water‐soluble drug that requires the help of the organic cation transporter (OCT2) to cross the cell membrane.^104^ OCT2 shows sexual dimorphic expression in many tissues of many species, with higher expression in males,^105^ which is related to androgen regulation.^106^ However, Inyang et al thought the sex difference cannot be explained by metformin pharmacokinetics because metformin levels in the plasma and brain of female mice are higher than those of male mice.^14^ Metformin is the only AMPK activator with sex‐specific effects, sex differences may be owing to a link in the AMPK pathway. However, another paper does not support this view.^9^ On the contrary, they reported that metformin had the same effect on male and female mice after lumbar disc puncture, although mechanical pain was more severe in female mice before treatment.

According to a study of data from a UK biobank, T2DM patients are more likely to report musculoskeletal pain in shoulder, neck, knee, or hip, particularly among women.^107^ The development of musculoskeletal pain may be related to the production of AGEs, leading to an inflammatory metabolic environment and oxidative stress, reducing connective tissue elasticity and weakening tendons.^108^ A follow‐up study by the same team said diabetics with metformin were less likely to have musculoskeletal pain, especially in women.^109^ We did not find any other clinical studies on gender differences in metformin analgesia. Whether there is a gender difference in the analgesic effect of metformin needs further study.

## 5 DISCUSSION

Metformin is a first‐line treatment for T2DM patients, and it is also used to treat polycystic ovary syndrome, hyperlipidemia, coronary artery disease, obesity, and a variety of kidney diseases such as acute kidney diseases, diabetic kidney disease, urolithiasis, renal cell carcinoma, end‐stage renal disease, and renal fibrosis.^110^, ^111^ There is considerable evidence that metformin also has beneficial effects on neuroprotection and hyperalgesia.^5^, ^112^ Metformin can reduce inflammatory response and oxidative stress and regulate autophagy response and the activation or expression of NaV1.7 and TRPA1 channels to play analgesic and antihyperalgesia roles by activating AMPK. Metformin's analgesia is also partially explained by the reduction in MGO and IR. These results provide a certain basis for metformin to be a potential drug in the treatment of PDN.

However, there are discrepancies between clinical data and preclinical data. Only one study showed a lower frequency of neuropathy in patients using metformin,^90^ and others showed a similar^86^ or higher^89^, ^91^, ^94^, ^95^ incidence of neuropathy in metformin patients. Vitamin B12 deficiency caused by metformin was thought to be associated with a higher incidence of neuropathy. Studies have generally shown metformin was associated with lower plasma vitamin B12 concentrations,^113^, ^114^ but the association between metformin‐induced vitamin B12 deficiency and DN remains controversial because of confounding factors including age, study environment, duration of diabetes, and dose and duration of metformin in clinical studies. Neuropathy associated with vitamin B12 deficiency is difficult to distinguished from DPN in T2DM patients. Therefore, the conclusion that a higher frequency of neuropathy is associated with metformin‐induced vitamin B12 deficiency is controversial. It has also been reported that metformin reduces only the level of nonfunctional plasma vitamin B12 but increases vitamin B12 accumulation in the liver.^115^ Low plasma B12 levels in metformin‐treated patients do not reflect impaired B12 status but rather altered tissue distribution and metabolism of vitamin B12. Metformin was associated with improved intracellular vitamin B12 metabolism despite low plasma vitamin B12, which has been clinically confirmed. Compared with T2DM patients without metformin, patients on metformin have a comparable red blood cell B12, a slightly lower methylmalonic acid, and better methylation index despite a low B12 plasma status,^116^ which suggested that metformin treatment was associated with improved intracellular vitamin B12 metabolism. Intracellular vitamin B12, rather than plasma vitamin B12, acts as a cofactor for enzymes that play a critical role in neuroprotection,^117^ so metformin‐induced plasma vitamin deficiency may have little effect on the DPN.

Even though the association between diabetic neuropathy and metformin‐induced vitamin B12 deficiency is unclear, as a matter of best safety, we recommend regular screening for serum vitamin B12 levels and peripheral neuropathy in T2DM patients on long‐term metformin use. However, because of financial and medical constraints, regular testing of vitamin levels is a burden for many patients, and researchers have suggested that the Metformin Usage Index (MUI) be used as a risk assessment tool to assess vitamin B12 deficiency in T2DM patients. MUI is the product of the dose (mg) of metformin used and its duration (years) divided by 1000, and patients have a high risk of vitamin B12 deficiency when the MUI>5.^118^

There are still some limitations in our study. Contrary to the results of basic studies, metformin does not provide significant neuroprotection in patients with T2DM in clinical practice, which may be because of vitamin B12 deficiency. The clinical and basic researches have different concerns. Basic researches focused on metformin's neuroprotective effects and mechanisms, whereas clinical studies focused on vitamin B12 deficiency. The shorter duration of metformin intervention in the primary study may be responsible for the lack of reports of vitamin B12 deficiency and associated adverse events. Metformin combined with vitamin B12 may be beneficial for diabetic neuropathy. Vitamin B12 in combination with metformin in T2DM patients reduced the incidence of peripheral neuropathy compared with metformin alone,^119^ but the trial did not set a control group without metformin. Further clinical trials are needed to investigate the neuroprotective effects of metformin. In addition, most studies have been conducted in patients with type 2 diabetes, whereas metformin's effect on neuropathy in patients with type 1 diabetes is poorly understood.

In conclusion, the therapeutic effect of metformin on DPN has been confirmed in many basic studies, and more and more studies have been conducted on its mechanism. However, clinical studies have focused on whether metformin‐induced vitamin B12 deficiency exacerbates neuropathy. This contradiction is worthy of attention and research. More clinical studies are needed in the future to observe the efficacy and safety of metformin in the treatment of diabetic neuropathy.

## AUTHOR CONTRIBUTIONS

J.W. conducted the study and wrote the text. S.J. gave research and writing guidance and approved the final version. Yanling Wei revised grammer and technical terms to make the artical more accurate. Meiyan Huang and Peng Wang were the main producer of figures and tables.

## FUNDING INFORMATION

No external funding.

## CONFLICT OF INTEREST

The authors declare that they have no competing interest.
